# Reply – RE: Policy Versus Practice: Facilitators and Barriers of Chronic Care Integration in Dutch General Practice – a Survey Study

**DOI:** 10.5334/ijic.11051

**Published:** 2026-05-05

**Authors:** Toine E. P. Remers, Simone A. Van Dulmen, Erik W. M. A. Bischoff, Florien M. Kruse, Marcel G. M. Olde Rikkert, Patrick P. T. Jeurissen

**Affiliations:** 1 Radboud university medical center, Department of IQ Health, Nijmegen, Netherlands; 2 Department of General Practice, Erasmus MC, Rotterdam, Netherlands; 3 Radboud university medical center, Department of Geriatric Medicine, Nijmegen, Netherlands; 4 Radboud university medical center, Donders Institute for Brain Cognition and Behaviour, Department of Geriatric Medicine, Radboud Alzheimer Centre, Nijmegen, Netherlands

**Keywords:** multimorbidity, integrated care, primary care, care groups, health policy, general practitioners

Dear Editor,

We would like to thank the author(s) of the Letter to the Editor for their interest in our article published in the International Journal of Integrated Care and for their careful reading of our work.

While we understand the nuance raised by the author(s) regarding the interpretation of our survey results, we would like to emphasize that this is primarily a matter of perspective and that we made a deliberate and carefully considered choice in how we reported these findings. Delivering integrated care is complex and demanding, particularly within the context of chronic care management. Our study aimed to provide first insights and reflections into the experienced ability of primary care professionals to perform such chronic care management.

As correctly noted by the author(s) of the letter, 39% of respondents neither agreed nor disagreed with the statement about feeling capable of delivering integrated care. While this group indeed did not explicitly state that they felt incapable, they also did not state explicitly to feel confident in their ability to provide integrated care. We – supported by the peers who reviewed this article – interpreted this neutrality as indicative of doubt regarding one’s capacity to deliver integrated care, and therefore as an expression of the current limitations experienced in practice. We felt supported in this rationale by the way in which such uncertainty about one’s ability to perform a task is also effectively being treated as a reason to refrain from action until that uncertainty is resolved in many other professional domains. For example, a surgeon who is unsure about successfully completing an operation is likewise expected not to perform it until they are confident in a positive outcome, and a pilot who doubts their ability to safely conduct a flight does not proceed until that doubt is removed. The general adagium in medical care already for centuries is: in dubio abstine. Most physicians will still shape their willingness to deliver new interventions including (the more laborious) integrated care also along this headline. For this reason, we deliberately phrased our findings in the abstract and results section as indicating that 56% of respondents did not explicitly report feeling capable of delivering integrated care. Readers can further nuance or interpret these findings from their own perspectives based on the details we have provided in Table 2, as the writer(s) of this letter has done as well.

Regarding the request to provide more detailed insights into the results by professional subgroup, we certainly understand the value of such detailed insights. However, the primary aims of our study required us to capture overarching insights from a broad and representative sample of healthcare providers working in Dutch general practices leading to high-level experiences and perceptions across the field as a whole. Subgroup-specific interpretations reflect a complex interplay of professional roles, expectations, and contextual factors, and we believe these would be more appropriately explored in dedicated follow-up studies focused on identifying explanatory mechanisms within each discipline, rather than as additional descriptive statistics within the current study.

In closing, we are grateful for the thoughtful response to our work and for the opportunity to further clarify our methodological and interpretative choices. We hope that this explanation helps to illuminate the rationale behind how we presented our findings and contributes to continued constructive discussion on the challenges of integrated chronic care in general practices.

Kind regards,

Dr. Toine E.P. Remers

Dr. Simone A. Van Dulmen

Prof. Dr. Erik W.M.A. Bischoff

Dr. Florien M. Kruse

Prof. Dr. Marcel G.M. Olde Rikkert

Prof. Dr. Patrick P.T. Jeurissen

      https://ijic.org/articles/10.5334/ijic.8443

